# Implementation of lifestyle recommendations in the Dutch medical specialists’ guidelines: a mixed-methods cross-sectional survey study

**DOI:** 10.1136/bmjopen-2026-120063

**Published:** 2026-08-10

**Authors:** Florien Ham, Anh Nhi Nguyen, Jolanda Keijsers, Elisabeth van Rossum, Mariette Boon, Janke de Groot

**Affiliations:** 1 Knowledge Institute, Dutch Association of Medical Specialists, Utrecht, Netherlands; 2 Department of Internal Medicine, Erasmus MC University Medical Center, Rotterdam, Netherlands; 3 TNO Prevention and Health, Leiden, Netherlands

**Keywords:** Guideline Adherence, Delivery of Health Care, Integrated, Patient Participation, Attitude, Health Education

## Abstract

**Abstract:**

**Introduction:**

We recently showed that 65% of medical specialists’ guidelines in the context of chronic disease management in the Netherlands contain recommendations on lifestyle. However, it remains unclear how these recommendations are perceived and implemented in clinical practice.

**Objectives:**

To analyse (1) the level of implementation of these recommendations and (2) the knowledge, attitudes and needs to further facilitate the implementation of lifestyle recommendations in chronic disease management within specialist care from both patient and healthcare professional perspectives.

**Design:**

Mixed-methods cross-sectional study using two surveys.

**Setting:**

Hospital care in the Netherlands.

**Participants:**

Medical specialists affiliated with the Dutch Association of Medical Specialists (n=193) and patients who had visited a medical specialist within the previous 6 months, recruited via the Dutch Patient Federation (n=1057).

**Exposure:**

Lifestyle recommendations embedded in clinical practice guidelines for chronic disease management.

**Main outcome measures:**

The level of implementation is assessed by the self-reported frequency of lifestyle discussions and familiarity with lifestyle recommendations in clinical practice guidelines.

**Results:**

Patients and medical specialists report different levels of implementation. While 72% of medical specialists reported discussing lifestyle topics during consultations, only 37% of patients recalled such discussions. Guideline familiarity among medical specialists was significantly associated with applying lifestyle advice in clinical practice (Fisher’s exact test, p=0.02). Both groups expressed positive attitudes toward addressing lifestyle as part of medical specialist care consultations. Although medical specialists considered the lifestyle recommendations applicable, they identified key factors for successful implementation, including the framing of recommendations, patient motivation and capacity to make lifestyle changes and the organisational and societal contexts in which lifestyle recommendations are delivered.

**Conclusions:**

Although both medical specialists and patients recognise the importance of addressing lifestyle in healthcare, implementation in clinical practice can still be improved. Strengthening implementation requires institutions to allocate adequate resources to support these consultations, including protected time, targeted training and clearly defined referral pathways. Broader societal and political commitment, ensuring that prevention becomes a priority and shared responsibility across the healthcare ecosystem.

**STRENGTHS AND LIMITATIONS OF THIS STUDY**

- The two-sided approach, combining perspectives from medical specialists and patient-reported experiences, enabled identification of both overlaps and discrepancies in reported lifestyle discussions.
- The mixed-methods design, integrating quantitative and qualitative data, strengthened the robustness and depth of the findings.
- Participation among medical specialists was limited, which may affect the representativeness of the findings and suggests potential selection bias.
- Self-reported data and possible social desirability bias may have led to an overestimation of implementation levels and positive attitudes towards lifestyle discussions.

## Introduction

 The growing prevalence of chronic conditions impacts healthcare systems globally, mainly by the increased healthcare needs associated with chronic conditions.^1^ These needs do not only substantially impact healthcare burden and capacity but also healthcare expenditures.^2^ Moreover, in the context of an ageing society, the prevalence of patients living with multiple chronic conditions continues to rise.^3^

Guidance for managing (multiple) chronic conditions is described in clinical practice guidelines (CPGs). From these CPGs, lifestyle recommendations are a fundamental part of treatment, also within hospital care settings. For example, in CPGs for treating hypertension,^4^ depression^5^ and cardiovascular disease,^6^ lifestyle recommendations have a prominent position. In addition, in a cross-sectional analysis from the Dutch CPGs, we previously showed that lifestyle recommendations were included in 65% of the CPGs addressing chronic conditions.^7^

CPGs are important tools for the implementation of lifestyle innovations.^8^ However, implementing lifestyle recommendations seems a complex endeavour, requiring structural behavioural changes from both medical specialists and patients.^9 10^

It remains unclear how and which key determinants are related to implementation of lifestyle recommendations in hospital care settings. Therefore, the goal of this study was to analyse the current adoption of lifestyle recommendations in daily practice and to identify key factors supporting or limiting implementation of lifestyle recommendations in hospital care to support the implementation process. In this context, this study is using the Consolidated Framework for Implementation Research (CFIR) as a theoretical framework. The CFIR provides a comprehensive taxonomy of constructs that influence implementation across five domains: (1) intervention characteristics, (2) outer setting, (3) inner setting, (4) characteristics of individuals and (5) process. Its structured approach allows for systematic identification of determinants relevant to the adoption and integration of evidence-based practices.^11 12^

## Methods

### Study design and setting

A cross-sectional survey design was employed to examine the level of implementation of and the needs and attitudes towards lifestyle recommendations in Dutch CPGs for medical specialists. Two online surveys were developed in Microsoft Forms: the primary survey targeted medical specialists, while the secondary survey targeted patients, serving as a mirror to the specialists’ data on implementation and attitudes. This cross-sectional study was conducted between August and October 2024 in the Netherlands as part of a larger project from the Dutch Lifestyle in Healthcare Consortium beyond the scope of medical specialist care only. An English language version of the surveys can be found in the online supplemental material.

The study was guided by the CFIR, which was used to develop and analyse the questionnaire and to identify and structure factors relevant to the implementation of lifestyle recommendations in medical specialist care.^11^

This study focuses on nine disease categories, based on existing disease rankings by the Dutch National Institute for Public Health and the Environment, which consider prevalence, years of (healthy) life lost and disease burden. The included diseases consisted of cancer, cardiovascular diseases, respiratory diseases, depression, obesity, skin diseases, rheumatic diseases, diabetes mellitus (type 1 and 2) and dementia. This selection aligns with the analysis of lifestyle recommendations in CPGs conducted prior to this study.^7^

Throughout the larger project, ‘Lifestyle intervention’ was defined as ‘promoting sustained behavioural change in one or more of the following domains: physical activity, smoking, alcohol consumption, nutrition, relaxation and sleep’. This definition corresponds to the six pillars of lifestyle medicine defined by the American College of Lifestyle Medicine.^13^

### Survey development

To guide the development of the two questionnaires assessing the implementation of lifestyle recommendations from CPGs, the CFIR^11 12^ was used as a sensitising construct. The researchers (FH, AN, JK, JdG) selected the CFIR domains most relevant to the study goal, including the first domain (intervention characteristics) and the fifth domain (process). The selected constructs were translated to questions for the surveys. For the medical specialists’ survey, these questions concerned the application of lifestyle advice in clinical practice, knowledge and experience with lifestyle recommendations from clinical guidelines, as well as attitudes toward and satisfaction with the integration of lifestyle interventions in healthcare settings.

The level of implementation of lifestyle recommendations was assessed from both medical specialist and patient perspectives. Medical specialists were asked to what extent they discussed lifestyle with their patients during consultations, using a Likert scale, stratified by their familiarity with lifestyle recommendations from CPGs. Patients were asked whether lifestyle had been addressed by their treating medical specialist during consultations (yes/no).

Attitudes toward lifestyle discussions were assessed for both medical specialists and patients by rating the perceived importance, satisfaction and pleasantness of these discussions on Likert scales. Needs and experiences related to the implementation of lifestyle recommendations were primarily assessed among medical specialists using open-ended questions.

The initial drafts of both surveys were developed by four designated researchers (FH, AN, JK, JdG). The surveys included a mix of multiple-choice, Likert-scale and open-ended questions to capture both quantitative and qualitative insights. The survey was designed such that all questions required for the analyses had to be completed before submission. Prior to distribution, the survey underwent a pre-testing phase with a working group of the Lifestyle in Healthcare Consortium to gain feedback on clarity, wording and usability during an interactive working session to minimise response error. After the working session, the surveys were revised based on the feedback provided. Additionally, the surveys were tested within Microsoft Forms to ensure proper functionality and logical flow. The final versions of the surveys were approved by the research team before distribution.

### Participants

A non-probability convenience sampling approach was used. For this study, data from healthcare professionals were eligible if they 1) were a medical specialist (in training), 2) signed informed consent to use their data anonymously and 3) treated at least one of the preselected disease groups. Healthcare professionals were recruited by an online invitation to fill in the survey posted in the newsletter from their scientific societies or personally by e-mail. Furthermore, a news item, including an invitation to the survey, was posted on the news page of the Dutch Association of Medical Specialists.

Data from patients were eligible if they 1) visited a medical specialist in the previous 6 months for at least one of the preselected disease groups and 2) signed informed consent to use their data anonymously. Patients were recruited online through an existing research panel from the Dutch Patient Federation with 20.000 panel members.

In addition, both participant groups (medical specialists and patients) were recruited through LinkedIn. Participation was voluntary and based on self-selection, and no incentives were offered. No formal a priori sample size calculation was performed because no reliable estimate of the number of eligible participants or expected response rate was available. Given the exploratory nature of the study and the use of non-probability convenience sampling, all eligible participants who completed the survey during the recruitment period were included. Responses were screened for potential duplicate entries based on available identifiers prior to analysis. No duplicate responses were identified.

### Data analysis

Data were collected in Microsoft Forms and then transported to Microsoft Excel V.16.92 (Microsoft Corporation, 2021). Data were checked for completeness and consistency before analysis. No response errors or implausible values requiring correction or exclusion were identified. Descriptive statistics were used to analyse the characteristics of the respondents. For the analysis of the answers to open questions concerning the needs and experiences in current practice, we applied thematic qualitative analysis. The answers were coded using a deductive thematic content analysis by applying the CFIR domains and associated constructs as a predefined framework. The analysis focused on the first four CFIR domains, while the implications for the fifth domain (process) are addressed in the interpretation of the findings.

During interactive working sessions, three researchers (FH, AN and JdG) applied open coding to establish themes that emerged from the data. During this process, the codebook was constantly updated until a final coding tree could be established.

### Sensitivity analyses

For sensitivity analyses, we assessed whether familiarity with lifestyle recommendations in CPGs was associated with the extent to which healthcare professionals discussed lifestyle with their patients. Familiarity with guidelines was assessed using a dichotomous item (‘familiar’ vs ‘not familiar’). The extent to which lifestyle recommendations were applied in clinical encounters was measured using a 5-point Likert scale ranging from ‘(almost) never’ to ‘with (almost) all patients’. The associations were explored using a contingency table, followed by a Fisher’s exact test with Monte Carlo simulation (10.000 replicates) to obtain a robust estimate of the strength and significance of the association. All analyses were conducted in R (V.2025.09.2). The Likert categories were analysed in their full ordinal form. The results were presented using descriptive proportions and a Likert-style bar figure, providing a visual comparison of how frequently lifestyle recommendations were applied across the two knowledge groups. A two-sided p value < 0.05 was considered statistically significant.^14^

## Results

### Survey response

#### Medical specialists

A total of 525 healthcare professionals started the cross-sectional survey, of whom 402 completed all survey questions, resulting in a completion proportion of 76.6%. After applying eligibility criteria, 193 healthcare professionals were included in the analyses (online supplemental figure S1). Respondents were distributed across all 12 provinces, resulting in a geographically representative sample of the Netherlands (data not shown). Most responding medical specialists were female (67%) and 70% had over 11 years of professional experience. A variety of disciplines were represented, of which internists were the most prevalent (25%). The most prevalent disease groups medical specialists mentioned to treat in daily practice were cancer (46%), overweight/obesity (42%) and cardiovascular disease (39%).

#### Patients

A total of 1493 patients started the cross-sectional survey, of whom 1383 completed all survey questions, resulting in a completion proportion of 92.6%. After applying eligibility criteria, 1057 patients were included in the analyses (online supplemental figure S2). Respondents were distributed across all 12 provinces, ensuring a geographically representative national patient sample (data not shown). Overall, 52% of patients were female, and the majority were in the higher age categories (aged over 60 years). Cardiovascular diseases were the most common (23%), followed by diabetes mellitus (15%), respiratory diseases (14%) and cancer (13%).

Table 1 shows an overview of the characteristics.

**Table 1:** Characteristics of the respondents (medical specialists and patients)

Characteristic	Medical specialists (n=193)	Patients (n=1057)
Gender		
Male	60 (31%)	503 (48%)
Female	129 (67%)	548 (52%)
Other	4 (2%)	2 (<1%)
Age (years)		
18<40		20 (2%)
40<60	n/a	210 (20%)
60<70		370 (35%)
≥70		457 (43%)
Professional experience (years)*		
0≤5	19 (10%)*	
6≤10	39 (20%)	n/a
11≤20	74 (38%)	
>20	61 (32%)	
(Treating) disease groups		
Cancer	88 (46%)	267 (25%)
Diabetes	25 (13%)	247 (23%)
Cardiovascular	75 (39%)	431 (41%)
Overweight/obesity	82 (42%)	109 (10%)
Pulmonary	44 (23%)	281 (27%)
Rheumatic	51 (26%)	244 (23%)
Skin	39 (20%)	18 (2%)
Dementia	23 (12%)	7 (<1%)
Specialism		Medical specialist visited
Cardiologist	· 9 (5%)	· 292 (28%)
Pulmonologist	· 10 (5%)	· 213 (20%)
Dermatologist	· 7 (4%)	· 110 (10%)
Internist	· 48 (25%)	· 220 (21%)
Gastroenterologist	· 5 (3%)	· 73 (7%)
Rheumatologist	· 20 (10%)	· 159 (15%)
Geriatrist	· 9 (5%)	· 3 (<1%)
Urologist	· 6 (3%)	· 104 (10%)
Surgeon	· 5 (3%)	· 134 (13%)
Orthopaedist	· 3 (2%)	· 10 (1%)
Sports physician	· 1 (<1%)	· 7 (<1%)
Gynaecologist	· 13 (7%)	· 25 (2%)
Otolaryngologist	· 0 (0%)	· 44 (4%)
Ophthalmologist	· 9 (5%)	· 87 (8%)
Rehabilitation doctor	· 8 (4%)	· 35 (3%)
Psychiatrist	· 1 (<1%)	· 30 (3%)
Oncologist	· 10 (5%)	· 75 (7%)
Anaesthesiologist	· 2 (1%)	· 23 (2%)
Emergency doctor	· 0 (0%)	· 4 (<1%)
Microbiologist	· 0 (0%)	· 2 (<1%)
Radiologist	· 0 (0%)	· 11 (1%)
Allergist	· 2 (1%)	· 0 (0%)
Plastic surgeon	· 13 (7%)	· 0 (0%)
Paediatrician	· 12 (6%)	· 0 (0%)

* Both patients and medical specialists could fill in more than one disease group of which 17 medical specialists were in training.

### Level of implementation of lifestyle in clinical practice

#### Implementation of lifestyle discussions

Of all medical specialists included, 139/193 (72%) reported discussing lifestyle with the majority or with (almost) all their patients during consultation, regardless of whether they were or were not familiar with lifestyle recommendations from CPGs. However, only 391/1057 (37%) of the patients indicated that lifestyle was addressed by their treating medical specialist.

**Figure 1:**
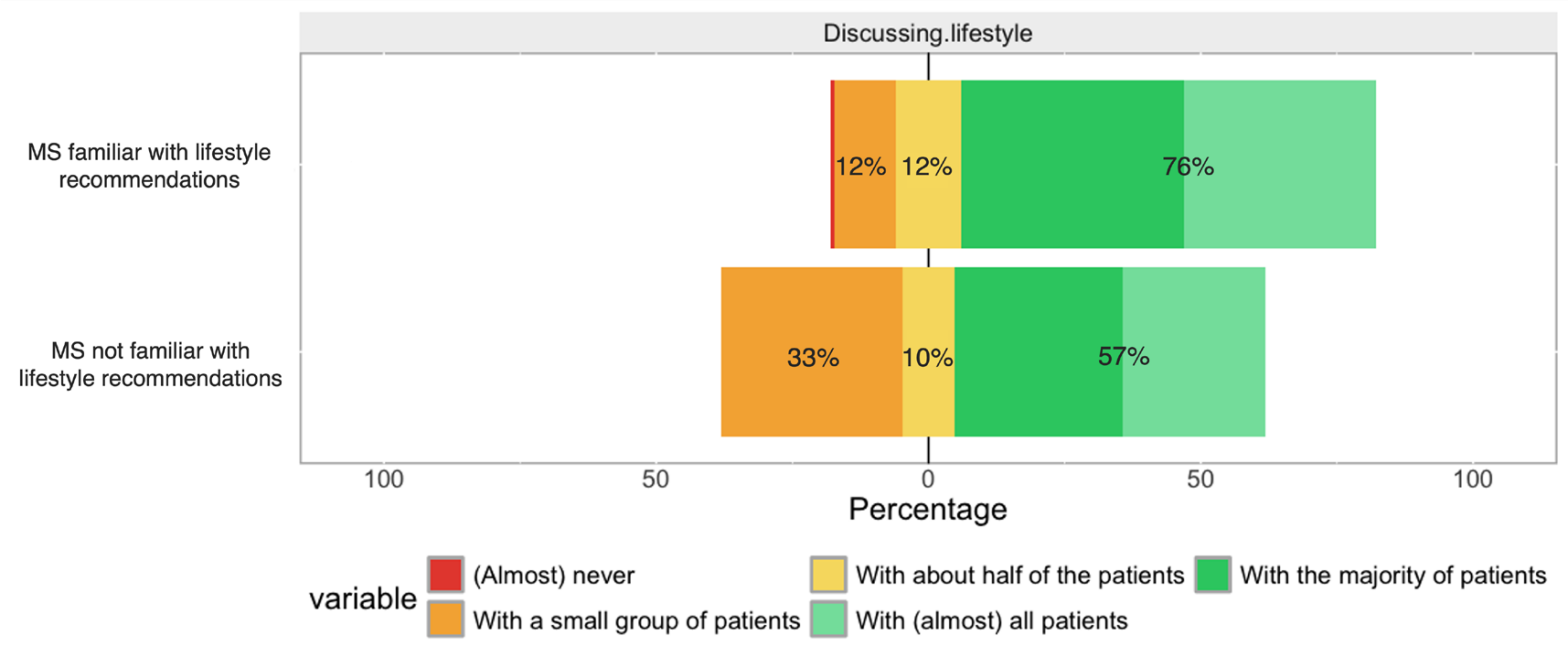
Frequency of lifestyle discussions according to familiarity with lifestyle recommendations. Responses were rated on a 5-point Likert scale, ranging from (*almost) never* to *with (almost) all patients*. Abbreviations: MS, medical specialists (in training).

As shown in figure 1, medical specialists who were familiar with lifestyle recommendations (n=151/193; 78%) reported discussing lifestyle more frequently in clinical practice compared with those who were not familiar with the guidelines (n=42/193; 22%). Fisher’s exact test demonstrated a statistically significant difference in the distribution of discussing lifestyle across the two groups (p=0.02), indicating that familiarity with guideline recommendations is associated with a higher likelihood of applying lifestyle advice in practice.

#### Implementation of lifestyle recommendations from CPGs

**Figure 2:**
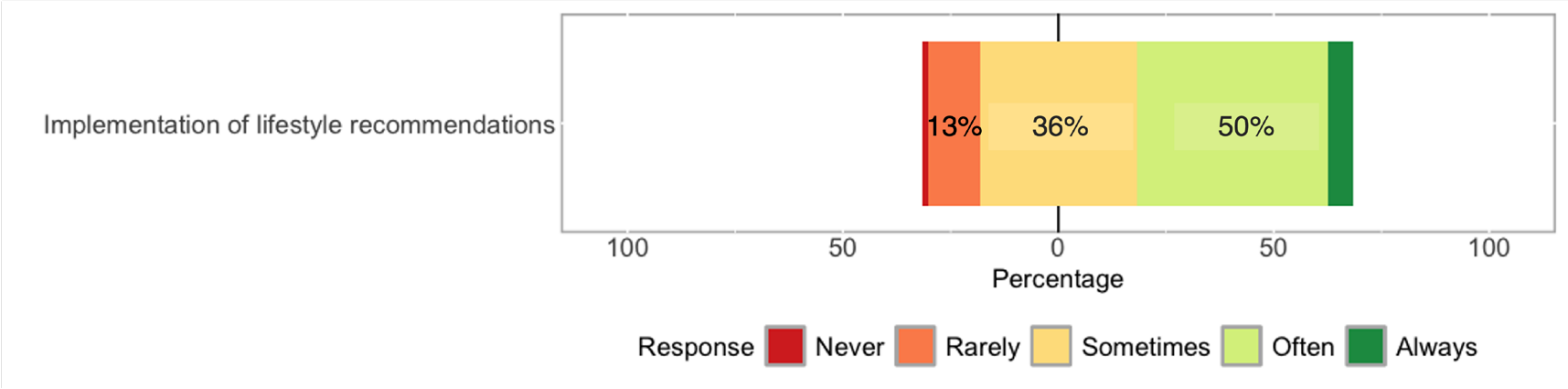
Implementation of lifestyle recommendations in clinical practice. The level of implementation was rated on a 5-point Likert scale, ranging from *never* to *always*.

Of all medical specialists, 151/193 (78%) were familiar with lifestyle recommendations from CPGs. Of this group, 75/151 (50%) of medical specialists reported applying them *always* or *often* in clinical practice (see figure 2).

#### Lifestyle factors discussed during consultations

**Figure 3:**
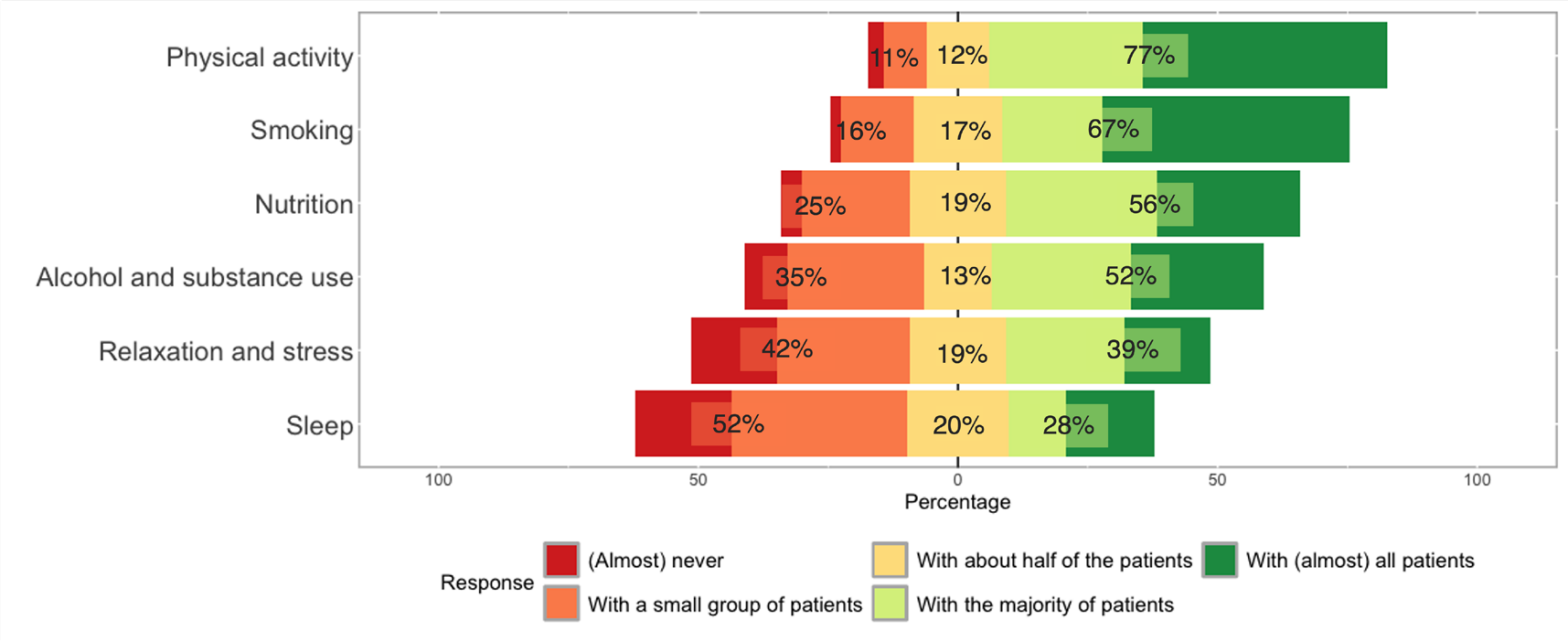
Lifestyle factors discussed during consultations, according to medical specialists. Responses were rated on a 5-point Likert scale, ranging from (*almost) never* to *with (almost) all patients*.

**Figure 4:**
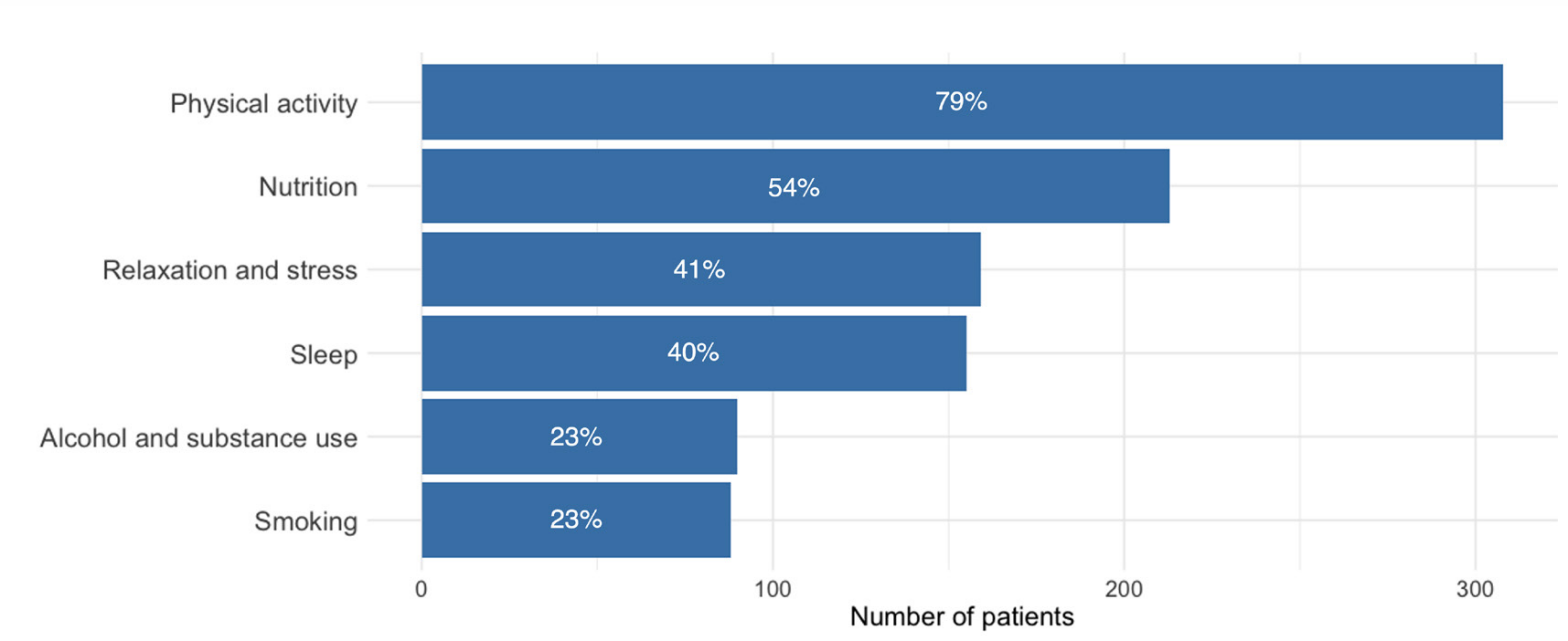
Lifestyle factors discussed during consultations, according to patients. The x-axis shows the number of patients indicating the topics being discussed with their treating medical specialists. Percentages indicate the proportion of patients endorsing each lifestyle factor. Totals exceed 100% because patients could select multiple factors.

Across all medical specialists in our study, physical activity emerged as the lifestyle factor most frequently discussed in clinical practice, regardless of their familiarity with lifestyle recommendations from CPGs. Similarly, patients reported that physical activity was the factor most often addressed during consultations. For the other lifestyle factors, reports varied substantially between medical specialists and patients. Figures 3 and 4 visualise the frequency with which each lifestyle factor was discussed according to medical specialists and patients, respectively.

### Factors affecting implementation lifestyle recommendations

**Figure 5:**
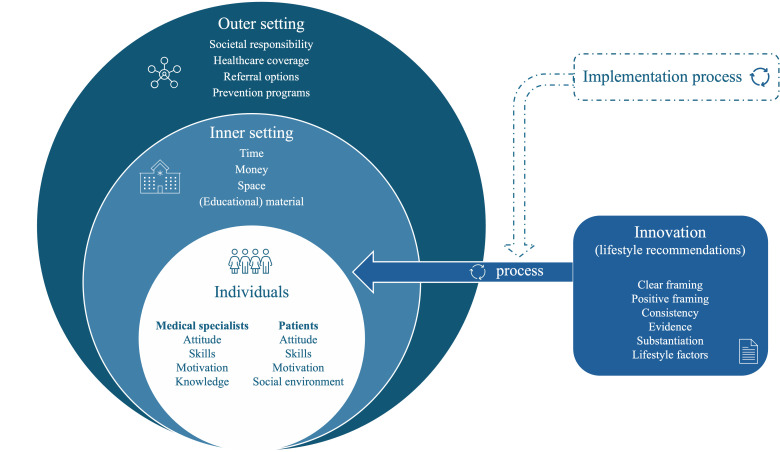
Summary of key factors affecting implementation of lifestyle in specialists’ daily practice. Factors are categorised by the domains of the CFIR framework for implementation. CFIR, Consolidated Framework for Implementation Research.

The factors affecting the level of implementation of guideline recommendations were assessed among the subgroup of medical specialists that were familiar with lifestyle recommendations in CPGs (n=151). As patient data could not be selected in this way, all patients were included in these analyses (n=1057). The factors were categorised according to the CFIR domains (*Innovation, Characteristics of the Individuals, Inner Setting* and the *Outer setting*) and elaborated by a combination of quantitative and qualitative data obtained from the survey. The implications for the fifth domain (*Process*) are addressed in the interpretation of the findings. Within the CFIR domains, the most prominent factors arising from the surveys are illustrated in figure 5 and further discussed in the following paragraphs.

#### Innovation (lifestyle recommendations)

The *Innovation* domain reflects the lifestyle recommendations in CPGs. According to 65% of the medical specialists, lifestyle recommendations were useful in providing lifestyle advice to their patients. A key factor affecting the usefulness of lifestyle recommendations included clear framing and consistency between different guideline recommendations about the same topic.

To gain a better understanding of the desired framing of lifestyle recommendations, we presented a selection of five differently framed recommendations and assessed the level of applicability. Based on the results, we could not distinguish favourably framed recommendations (online supplemental figure S3). However, based on the thematic analysis of the open-ended questions about applicability in our survey, we found that positive, clear and non-judgemental framing provides more guidance for clinical practice. Furthermore, substantiated recommendations, by scientific rationale and/or medical reasoning, contribute to embedding lifestyle recommendations into clinical practice. Key quotations are summarised in box 1.

Box 1 **Key quotations from medical specialists substantiating the applicability of lifestyle recommendations.**

*“*It provides guidance and stability.”

“It is good if there is a basis and patients do not receive different advice from everyone.”

“I find them too old-fashioned: judgmental.”

“Easy to explain and understandable for patients and easy to apply.”

“Sufficient evidence that there is an approach that is feasible for a sufficiently large group of people and that also has a clinical effect in the long term.”

#### Individuals (healthcare professionals and patients)

**Figure 6:**
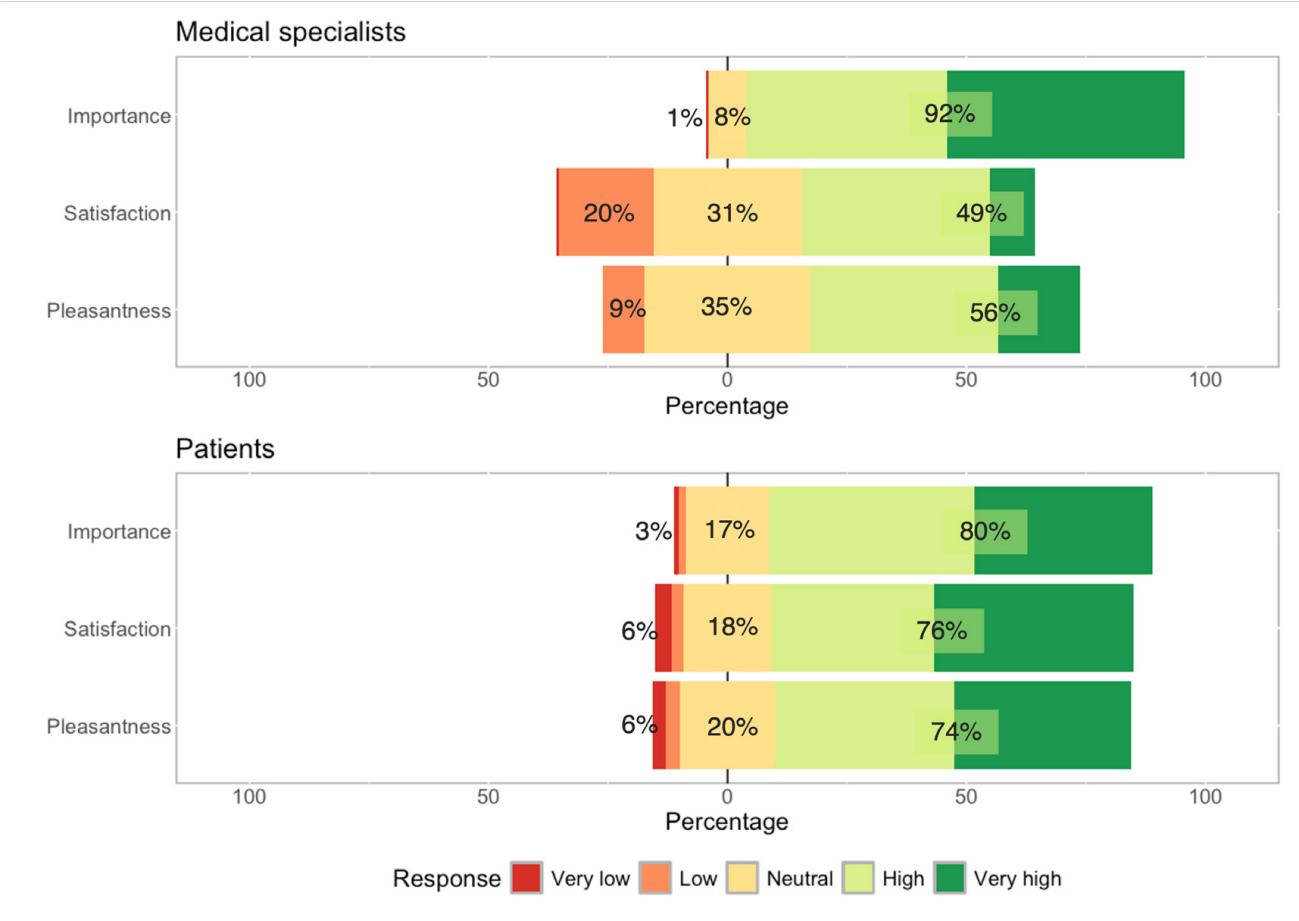
Attitudes of medical specialists and patients towards lifestyle discussions. Results are divided by three dimensions: importance, satisfaction and pleasantness. Each dimension was rated on a 5-point Likert scale, ranging from *very low* to *very high*.

The *Individuals* domain reflects both the medical specialists’ and the patients’ attitudes towards lifestyle discussions (see figure 6). Attitudes were assessed using a 5-point Likert scale across three dimensions: importance, pleasantness and satisfaction. Overall, both groups reported high to very high scores on all dimensions. However, ratings differed slightly: patients reported greater satisfaction and pleasantness with lifestyle-related care, whereas medical specialists rated the importance of lifestyle advice higher than patients.

Based on the thematic analysis of the open-ended questions on medical specialists’ attitudes, we identified several personal factors that appeared to influence both the level of implementation and attitudes towards lifestyle discussions, including personal beliefs, existing knowledge about lifestyle recommendations, perceptions of patients’ social context and stigmatisation. Key quotations are presented in box 2.

Box 2 **Key quotations from medical specialists substantiating attitudes towards lifestyle discussions.**

“Advice on behaviour change is pretty pointless.”

“The recommendations are of personal interest to me, so if it comes up in a medication consultation, I would like to explain it.”

“Patients appreciate the insight.”

“Every person is unique; we look at what is possible for each patient.”

“Sometimes absolute nonsense, many know this very well themselves.”

“How to deal with the stigmatizing effect of being overweight? - this often prevents healthcare providers from addressing the topic.”

“It is good to use evidence-based guidelines as a starting point to assess current behaviour and to develop a feasible plan together through shared decision-making.”

“Don't want to be too patronizing either.”

“Depending on the patient, this can sometimes be perceived as patronising, especially if there is already some nutritional knowledge available.”

#### Inner setting (hospital)

The *Inner setting* domain reflects the factors within the hospital that influence the level of implementation. Thematic analyses revealed that in this domain limited resources, including financial means, time, access to (educational) materials and physical space needed to apply lifestyle recommendations in clinical practice, were experienced as a key barrier for implementation by medical specialists. Key quotations are presented in box 3.

Box 3 **Key quotations from medical specialists substantiating limited resources as a barrier for implementation.**

*“*More referral options for patients, where they are followed to change their behaviour. No one-time advice. This can sow a seed or increase ambivalence, but it doesn't necessarily lead to behavioural change.”

“These are so many aspects of lifestyle that it is really difficult to fit it into a short consultation.”

**Figure 7:**
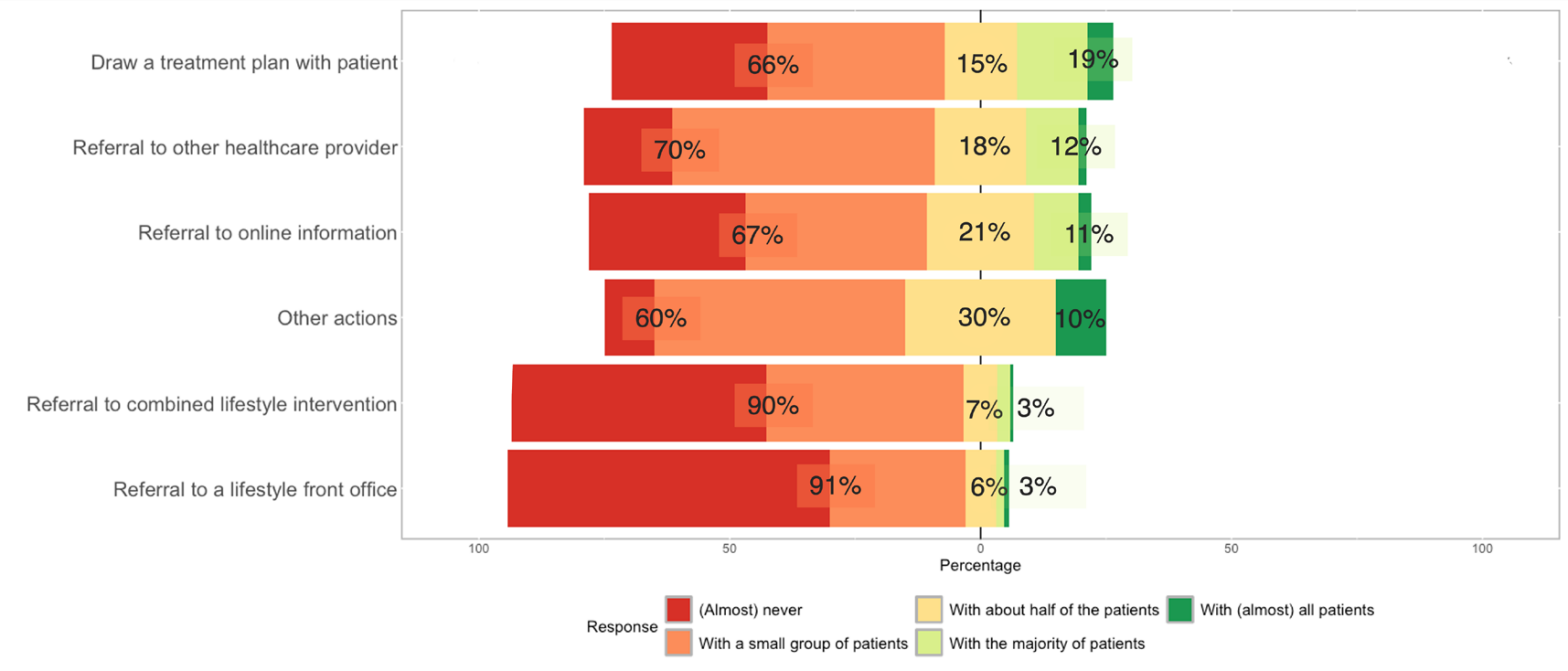
Subsequent actions taken after addressing lifestyle during consultations, according to medical specialists. Each action was rated on a 5-point Likert scale, ranging from (*almost) never* to *(almost) all patients*.

Additionally, most medical specialists did not undertake subsequent actions after addressing lifestyle during consultations (see figure 7). Similarly, 61% of patients mentioned adopting lifestyle changes independently, while 8% mentioned setting up a treatment plan with their treating medical specialists. Patient results are presented in online supplemental figure S4).

#### Outer setting (community, society, public domain)

The *Outer domain* reflects the factors outside of the hospital. Thematic analyses of the open-ended responses identified several factors influencing implementation. These included the need for greater societal responsibility, such as clear hospital policies on prevention and supportive political and public health initiatives. Medical specialists emphasised that promoting healthy behaviours should be a shared responsibility, as neither they nor their patients can address this alone. Key barriers included limited awareness of referral options outside the hospital and insufficient coverage by healthcare insurers. Key quotations are presented in box 4.

Box 4 **Key quotations from medical specialists reflecting the outer domain factors affecting the level of implementation.**

“Exercise groups are currently run on a voluntary basis; reimbursement from the health insurer would give me a positive boost and ensure future-oriented care.”

“A feeling that as a society we really strive for more healthy behaviour and that we do not give patients the feeling that it is an individual choice.”

“Lifestyle goes beyond healthcare provider and patient, for example supermarkets, food prices, city layout, politics.”

## Discussion

With the increasing role of medical specialists in lifestyle care in the context of chronic disease management, the purpose of the study was to evaluate the level of implementation of lifestyle recommendations in CPGs, as well as the needs among and the attitude regarding these recommendations. We applied a comprehensive approach, including perspectives from both medical specialists and patients and using surveys including closed- and open-ended questions. With our surveys, we identified several key factors affecting the level of implementation by categorising these factors in four domains according to the CFIR framework. These factors included patients’ and specialists’ attitudes towards and knowledge about lifestyle, the availability of resources to integrate lifestyle care into clinical practice, the broader societal context in creating a supportive environment for healthy behaviours and the presence of clear and actionable recommendations in CPGs. In the discussion we reflect on what these results mean to the implementation process (fifth domain of the CFIR framework).

Results showed the level of implementation of lifestyle in clinical practice was 72%, according to the medical specialists. In addition, we found a positive association between guideline familiarity and lifestyle discussions across medical specialists. This aligns with findings by Bolbrinker *et al.*^15^, who reported a positive association between guideline awareness and the level of implementation in a previous study of the European guidelines in the context of hypertension.^15^ In contrast, the level of implementation reported by patients was substantially lower (37%). This discrepancy may suggest differences in perception between medical specialists and patients regarding whether lifestyle is addressed during consultations. However, as data from medical specialists and patients were not matched at the individual level, alternative explanations cannot be excluded. Future studies using matched medical specialist–patient data could provide greater insight into these differences. Moreover, only half of the medical specialists reported applying lifestyle recommendations always or often in routine care, indicating that the consistent integration of guideline-based lifestyle recommendations into medical specialist practice could be further strengthened.

Overall, our findings correspond to the previously obtained core elements affecting implementation of guidelines in general, including the lack of knowledge or substantiation, patients’ perspectives, feasibility and own values and beliefs (attitudes).^16^ Importantly, this study adds to the existing literature by combining quantitative and qualitative data and by incorporating both medical specialist and patient perspectives, enabling a nuanced and multi-perspective assessment of the implementation of lifestyle recommendations in medical specialist care. Based on previous studies in this area, attitudes of both medical specialists and patients (the *individuals’* domain) towards lifestyle discussions seem to highly impact the level of implementation.^17^ Our results show that both medical specialists and patients recognised the relevance of lifestyle discussions in medical consultations, as the level of importance was rated (very) high. Pleasantness was perceived more positively by patients when compared with medical specialists, suggesting that patients may be more comfortable with these discussions than physicians anticipate. This is a good message to relay to those healthcare providers showing hesitation bringing up lifestyle due to ‘fear’ of patronising their patients in this and a previous study.^18^ Satisfaction showed the greatest discrepancy, with a higher satisfaction rate by patients when compared with medical specialists. These findings contradict previously published data, where one third of the patients indicated a low satisfaction with discussing lifestyle, even when feeling the need to address lifestyle topics.^19^ This discrepancy may partly be explained by differences in study populations, as our respondents recruited from the Dutch Patient Federation’s research panel were relatively highly educated and, by inclusion, positively biased towards lifestyle interventions. Furthermore, our qualitative findings indicated that some medical specialists felt uncomfortable discussing lifestyle because they feared being perceived as judgemental.

Next to the attitudes of the individuals, we investigated the usefulness of the lifestyle recommendations themselves (the *innovation* domain), as they do not always seem to be framed clearly, are often negative in tone and lack scientific substantiation, according to the medical specialists in our survey. In line with previous findings on lifestyle recommendations in the National Institute for Health and Care Excellence (NICE) guidelines and in the Dutch CPGs for medical specialists, clear framing and substantiation from scientific literature according to National Institute for Health and Care Excellence (GRADE) is often lacking.^7 20^ This calls for guideline developers to look at ways to improve both the wording of and the approaches used to appraise and incorporate different types of evidence, recognising that complex lifestyle interventions may require diverse and appropriate forms of evidence.^21 22^ Simultaneously, medical specialists did report the need for autonomy when it comes to lifestyle discussions. As lifestyle is a preference-sensitive topic for both medical specialists and patients, it necessitates a collaborative approach through shared decision-making (SDM).^23^ SDM is a collaborative approach where healthcare providers and patients make treatment decisions together, considering both scientific evidence and the patient’s individual preferences, values and goals.^24 25^ In light of SDM, medical specialists did emphasise the need to adapt communication to patients with limited health literacy and engage patients in value-driven conversations. The usefulness of lifestyle recommendations was also affected by some barriers from the hospital environment (the *inner setting* domain), including time, financial and spatial constraints. These barriers are not limited to lifestyle recommendations nor to medical specialists’ care.^26 27^ Medical specialists also highlight the need for political decisions and prevention programmes supporting public health and creating healthier environments,^28 29^ as the medical field cannot bear lifestyle-related chronic diseases alone. In line with this, a better connection with the social domain and community-based lifestyle initiative (the *outer setting* domain) will support implementation and facilitate better follow-up after referral.

### Strengths and limitations

This study has several important strengths. First, to our knowledge, it is the first to specifically examine the role and attitudes of medical specialists and patients towards lifestyle recommendations in the context of medical specialist care. This context is particularly relevant for chronic disease management, as medical specialists are uniquely positioned to integrate lifestyle recommendations into disease-specific treatment trajectories and follow-up.^30^ Second, the two-sided approach, combining perspectives from medical specialists with patient-reported experiences, enabled the identification of both overlaps and discrepancies in lifestyle discussions in practice. Finally, the use of a mixed-methods design strengthened the robustness and depth of our findings.

This study also has some limitations. First, participants were recruited using non-probability convenience sampling through an open survey link. Consequently, the number of healthcare professionals and patients reached was unknown, precluding calculation of response rates. Second, recruiting medical specialists for the survey was challenging, resulting in a relatively small sample size. This may reflect limited engagement with lifestyle-related topics and research within the medical field. In contrast, we were able to recruit a large group of patients from the Patient Federation’s research panel. The majority of this research panel includes patients in the higher age categories (aged 60 years or older). While this reflects the demographic profile of many patients with chronic conditions in medical specialist care, this may limit the generalisability of our results to younger patient populations. The results may therefore be somewhat biased, as healthcare providers and patients with greater interest in the topic were probably more likely to participate. In addition, self-reported data may have introduced recall and social desirability bias, potentially leading to an overestimation of implementation levels and positive attitudes among medical specialists, while barriers and needs for addressing lifestyle may be underestimated. Moreover, the qualitative assessment of barriers, facilitators and implementation needs in our study was mostly limited to the medical specialists’ perspective. Future research should further investigate both patient- and healthcare professional-related determinants of lifestyle implementation in clinical practice. Larger studies are needed to identify factors associated with whether medical specialists discuss and implement lifestyle recommendations, including potential differences between medical specialties. In addition, future research could provide more insight into the content and perceived impact of lifestyle discussions by incorporating qualitative data from patients.

### Future directions

Our findings highlight the urgent need for effective implementation strategies to support lifestyle recommendations in clinical practice. These strategies should be aimed at different domains within the CFIR framework to match with the implementation strategies from the CFIR Expert Recommendations for Implementing Change (ERIC) Implementation Strategy Matching tool. This tool combines concepts of CFIR with implementation strategies derived from the ERIC.^11 31^ At the level of the innovation, this study showed the importance of clear and evidence-based recommendations for optimising the uptake of the CPG’s in clinical practice in line with earlier research.^7^ On the individual level, structural embedding of lifestyle care requires investment in the education and training of medical specialists, ensuring that lifestyle counselling and referral skills are integrated into (postgraduate) medical education. Our quantitative findings on this are that lifestyle discussions do not consistently translate into concrete referrals or follow-up, highlighting the need for clear referral pathways and structured lifestyle support. One promising approach, addressing barriers at the inner level and quantitative findings on subsequent actions after discussion, is the recent introduction of so-called ‘Lifestyle Front Offices’ (LFOs) in Dutch hospitals.^32^ The LFO connects medical specialist care with community-based lifestyle and social support services by providing medical specialists with a clear and accessible referral point for lifestyle assessment and support. These developments may provide important strategic direction and facilitate nationwide implementation of lifestyle-oriented care models. To enable broader and sustainable uptake, future efforts should focus on rigorous evaluation of the LFO’s implementation processes and its impact on patient outcomes and healthcare system performance. Finally, our findings align with current developments on the policy level domain (outer setting) internationally, such as the WHO’s Global Action Plan on Physical Activity 2018–2030 and the WHO Global Guidelines on Physical Activity and Sedentary Behaviour.^33 34^

## Conclusion

This cross-sectional survey study showed that the current level of implementing lifestyle in clinical practice in the Netherlands seems reasonably high but relatively low from the patient perspective. This means that there is room for improvement by bridging this gap. Using a CFIR-informed approach, we identified several key determinants of implementation, including familiarity with guideline recommendations, appropriate framing of lifestyle discussions, clinicians’ attitudes and the availability of essential resources (time, funding and space). Together, these findings highlight that strengthening implementation efforts requires a multilevel strategy addressing the innovation itself (lifestyle recommendation) and the individuals’ and organisational conditions within healthcare settings, alongside better integration between healthcare and social care, such as hospital-based LFOs bridging medical and social domains.

## Supplementary material

10.1136/bmjopen-2026-120063 online supplemental file 1

## Data Availability

Data are available upon reasonable request.
