# Supplementary material for: Implementation of lifestyle recommendations in the Dutch medical specialists’ guidelines: a mixed-methods cross-sectional survey study
Source: BMJ Open. 2026 Aug 10;16(8):e120063. doi: 10.1136/bmjopen-2026-120063 (PMC13475326; doi:10.1136/bmjopen-2026-120063)
Supplement: Supplementary file 1 [file bmjopen-2026-120063.reviewer_comments.pdf]

PEER REVIEW HISTORY

BMJ Open publishes all reviews undertaken for accepted manuscripts. Reviewers are asked to complete a checklist review form and are provided with free text boxes to elaborate on their assessment. These free text comments are reproduced below.

ARTICLE DETAILS

Title (Provisional)

Implementation of lifestyle recommendations in the Dutch medical specialists' guidelines. A mixed-methods cross-sectional survey study.

Authors

Ham, Florien; Nguyen, Anh N.; Keijsers, Jolanda; van Rossum, Elisabeth; Boon, Mariette; de Groot, Janke

---

VERSION 1 - REVIEW

**Reviewer**	**1**
**Name**	**Asiri, Faris Y.**
**Affiliation**	**Department of Preventive Dental Sciences, College of Dentistry, King Faisal University**
**Date**	**15-Jun-2026**
**COI**	**None**

The manuscript addresses an important topic and demonstrates generally good adherence to STROBE recommendations for reporting cross-sectional studies. The study provides valuable insights into the implementation of lifestyle recommendations in specialist care. However, several aspects of reporting would benefit from further clarification to ensure closer alignment with STROBE recommendations and to further enhance the transparency and reproducibility of the findings. Additional details regarding sample size justification, the extent and handling of missing data, participant flow and response rates, reporting of effect estimates with corresponding precision measures (where applicable), and measures undertaken to minimise potential sources of bias would strengthen the methodological reporting of the study.

**Reviewer**	**2**
**Name**	**Pasman, Wilrike**
**Affiliation**	**TNO, Healthy Living**
**Date**	**16-Jul-2026**
**COI**	**None**

In the paper a straightforward evaluation of lifestyle recommendations of the CPG by medical specialists and according to patients is presented. It is a positive finding that medical specialists are aware of lifestyle recommendations for treatment. The fact that patients experience less lifestyle advice and no concrete action plans are provided stress that there is room for improvement with respect to lifestyle in health care as treatment of chronic disease. Concerns of the study are well mentioned.

Some remarks that could substantiate the paper with more details and content are listed below. Some topics could be included in the discussion to elaborate on it.

- The paper is very straightforward. Data is presented, especially for the medical specialists, and it seems that less focus on patients' perspectives is reported.
- Are perceptions and use of lifestyle advice for treatment depending upon the disease at hand? Was investigated which medical specialists used lifestyle recommendation more? What is the 22% of medical specialists that is not familiar with the lifestyle recommendations? Is their specialism known?
- It seems logical that only medical specialists participated who recognized this survey topic and therefore a selection bias is present as is mentioned in the limitations. The findings may therefore be specific for certain diseases and limit the generalizability to total health care.
- Specialists and patients clearly report different rates for discussion of lifestyle topics: 72 vs 37%. How come? Do patients not identify all factors as lifestyle of patients not able to understand all information provided? As mentioned by the authors, the rates cannot be compared directly since the patients were not the patients of the medical specialists in the study. This would be a nice follow-up to examine this in more detail.
- Was the CFIR method used the proper method? Speculate on this in Discussion. Was more information about facilitators and barriers obtained? Also, of patients? What happened with the fifth domain of the CFIR Process? No evaluation or results presented.
- An analysis of patient data with a similar background to the medical specialists may provide some more similarities in the lifestyle factors mentioned, because now data of patients with other diseases may dilute the data.
- Especially physical activity is the most discussed lifestyle factor, also mentioned by patients. Other factors differ in frequency and order of discussion; what could be an explanation for this? Awareness of the other lifestyle factors may be an issue for training for specialists and patients.
- For medical specialists it was found that about 20% found lifestyle recommendation less satisfying. What could be the reason for this?
- The results of Figure 7 illustrate that the medical specialists especially talk about lifestyle, but no actual concrete plans or referrals are provided. This is in line with the

findings of the patients that they feel they have changed their lifestyle themselves. This is a topic for discussion: how to enable specialists to become more concrete? How to support the patients? The LFO is a nice solution for this.

- It seems like less results are available from the patient survey. Even the questionnaire mentions 27 questions, but only 14 are shown in the supplement.
- The figure in the supplement is labeled opposite to the figures in the main paper (inconvenient with respect to reading).

VERSION 1 - AUTHOR RESPONSE

Reviewer 1 – Comments to the author

Dr. Faris Y. Asiri, Department of Preventive Dental Sciences, College of Dentistry, King Faisal University

The manuscript addresses an important topic and demonstrates generally good adherence to STROBE recommendations for reporting cross-sectional studies. The study provides valuable insights into the implementation of lifestyle recommendations in specialist care. However, several aspects of reporting would benefit from further clarification to ensure closer alignment with STROBE recommendations and to further enhance the transparency and reproducibility of the findings. Additional details regarding sample size justification, the extent and handling of missing data, participant flow and response rates, reporting of effect estimates with corresponding precision measures (where applicable), and measures undertaken to minimise potential sources of bias would strengthen the methodological reporting of the study.

We thank the editor for the careful evaluation of our manuscript and for the constructive comments. We appreciate the recognition that the manuscript addresses an important topic and generally adheres to the STROBE recommendations.

In response to the suggestions, we have revised the manuscript to improve the transparency and completeness. Specifically, we added the following:

- A description of the sampling approach and clarified that a formal sample size calculation was not performed because of the exploratory nature of the study and the use of convenience sampling;
- Flowcharts of inclusion (Supplementary Material Figure S1 and S2).
- Measures undertaken to minimise potential sources of bias, including pretesting of the survey to reduce response error;
- Measures undertaken to avoid duplicate entries, including the requirement that all questions needed to be completed before submission.

We believe these revisions improve the clarity, transparency, and reproducibility of the study and better align the manuscript with the STROBE reporting recommendations and with the Checklist for Reporting of Survey Studies (CROSS).

Reviewer 2 – Comments to the author

Dr. Wilrike Pasman, TNO

In the paper a straightforward evaluation of lifestyle recommendations of the CPG by medical specialists and according to patients is presented. It is a positive finding that medical specialists are aware of lifestyle recommendations for treatment. The fact that patients experience less lifestyle advice and no concrete action plans are provided stress that there is room for improvement with respect to lifestyle in health care as treatment of chronic disease. Concerns of the study are well mentioned.

We thank the reviewer for the positive evaluation of our manuscript and for recognizing the relevance of evaluating the implementation of lifestyle recommendations in clinical practice.

Some remarks that could substantiate the paper with more details and content are listed below. Some topics could be included in the discussion to elaborate on it.

We are grateful for the constructive suggestions to further strengthen the manuscript. In response, we have revised the manuscript by providing additional methodological details, clarifying several aspects of the analyses, and expanding the Discussion where appropriate to further substantiate the findings. Below, we address each comment in detail.

➢ The paper is very straightforward. Data is presented, especially for the medical specialists, and it seems that less focus on patients' perspectives is reported.
We thank the reviewer for this observation. The difference in the level of detail between the medical specialist and patient results reflects the design of the study. The questionnaire for medical specialists included additional questions on awareness, familiarity with, and implementation of guideline recommendations, as medical specialists are directly responsible for applying these recommendations in clinical practice. Patients, in contrast, are not expected to know whether guideline recommendations have been followed or the reasons underlying clinical decision-making. Therefore, the patient questionnaire was limited to their experiences of lifestyle discussions and referrals during appointments with their physician. We believe this complementary approach provides valuable insights from both perspectives. However, we agree that a detailed qualitative assessment of patients' perspectives on the barriers, facilitators, and support needs related to following up on lifestyle recommendations which was beyond the scope of the current study, indeed warrants further investigation (as mentioned in the Discussion).

➢ Are perceptions and use of lifestyle advice for treatment depending upon the disease at hand? Was investigated which medical specialists used lifestyle recommendation more? What is the 22% of medical specialists that is not familiar with the lifestyle recommendations? Is their specialism known?
Thank you for this interesting suggestion. We explored whether the use of lifestyle recommendations differed across medical specialties. Although no clear pattern was observed, the number of participants within each specialty was too small to allow meaningful statistical comparisons. Therefore, we cannot draw firm conclusions about differences between specialties. Medical specialists who reported not being familiar with the lifestyle recommendations represented a wide range of specialties, including internal medicine, ophthalmology, orthopaedics, anaesthesiology, pulmonology, dermatology, paediatrics, gastroenterology, gynaecology, plastic surgery, rheumatology, radiology, urology, surgery, and rehabilitation medicine. Therefore, we cannot draw firm conclusions regarding specialty-specific differences. We agree that this would be an interesting topic for future research. Therefore, we incorporated some suggestions for future research about this topic in the Discussion:
"Future research should further investigate both patient- and healthcare professional-related determinants of lifestyle implementation in clinical practice. Larger studies are needed to identify factors associated with whether medical specialists discuss and implement lifestyle recommendations, including potential differences between medical specialties. In addition, future research could provide more insight into the content and perceived impact of lifestyle discussions by incorporating qualitative data from patients."

➢ It seems logical that only medical specialists participated who recognized this survey topic and therefore a selection bias is present as is mentioned in the

limitations. The findings may therefore be specific for certain diseases and limit the generalizability to total health care.

We agree, and reflected on this in de discussion, that our sampling strategy (convenience sampling through online invitations, newsletters, and LinkedIn) could have introduced selection bias and may have limited the generalizability of our findings to the broader population of healthcare professionals. Whether our findings are disease-specific is difficult to determine, as our qualitative findings suggest that the implementation of lifestyle recommendations is influenced by multiple factors beyond the disease itself, including healthcare professionals' knowledge, attitudes, perceived roles and responsibilities, and organisational support. Due to the limited sample size within individual medical specialties, we were unable to statistically investigate whether participation or the use of lifestyle recommendations differed by specialty or disease area. We agree that this would be an interesting topic for future research (see copy of Discussion text in the previous comment).

➢ Specialists and patients clearly report different rates for discussion of lifestyle topics: 72 vs 37%. How come? Do patients not identify all factors as lifestyle of patients not able to understand all information provided? As mentioned by the authors, the rates cannot be compared directly since the patients were not the patients of the medical specialists in the study. This would be a nice follow-up to examine this in more detail.

We thank the reviewer for this thoughtful comment. We agree that the observed differences between medical specialists' and patients' reports warrant further investigation. We added a suggestion for future research about this topic in the Discussion: "Future studies using matched medical specialist–patient data could provide greater insight into these differences."

➢ Was the CFIR method used the proper method? Speculate on this in Discussion. Was more information about facilitators and barriers obtained? Also, of patients? What happened with the fifth domain of the CFIR Process? No evaluation or results presented.

We thank the reviewer for this relevant comment. We agree that this point required further clarification. First, CFIR was used as a conceptual implementation framework rather than as a research method. Although several implementation frameworks are available, we selected CFIR because it provides a comprehensive framework to identify implementation determinants across multiple levels and can be linked to implementation strategies, for example through the ERIC taxonomy. Our aim was to identify determinants that may influence the future implementation of LFOs rather than to evaluate the implementation process itself. Therefore, the fifth CFIR domain, *Process*, was not analysed as a separate domain. Instead, it was addressed by reflecting on how the identified determinants may inform the implementation process. We have clarified this in the revised manuscript as follows:

Methods: "The analysis focused on the first four CFIR domains, while the implications for the fifth domain (Process) are addressed in the interpretation of the findings."

Results: "The implications for the fifth domain (Process) are addressed in the interpretation of the

findings."

Discussion: "In the discussion we reflect on what these results mean to the implementation process (fifth domain of the CFIR framework)."

➢ An analysis of patient data with a similar background to the medical specialists may provide some more similarities in the lifestyle factors mentioned, because now data of patients with other diseases may dilute the data.
We agree that disease-specific analyses could provide additional insights. However, the sample sizes within individual disease groups and corresponding medical specialties were too small to allow meaningful statistical comparisons. Future studies with larger samples including matched patient populations and corresponding medical specialties would be valuable (see copy of Discussion text in the comments earlier).

➢ Especially physical activity is the most discussed lifestyle factor, also mentioned by patients. Other factors differ in frequency and order of discussion; what could be an explanation for this? Awareness of the other lifestyle factors may be an issue for training for specialists and patients.
We thank the reviewer for this interesting observation. We agree that physical activity was the lifestyle factor most frequently discussed by both medical specialists and patients. One possible explanation is that physical activity is often perceived as a relatively concrete and less sensitive topic to discuss compared with other lifestyle factors. In addition, physical activity has received considerable attention in clinical practice guidelines when compared to other lifestyle factors, as also described in our previous work. However, our study was not designed to explore the underlying reasons for these differences, and any further interpretation would be speculative. Therefore, we have not expanded on this point in the Discussion. We agree that future research could further explore whether awareness and knowledge of different lifestyle factors among both healthcare professionals and patients contribute to these differences.

➢ For medical specialists it was found that about 20% found lifestyle recommendation less satisfying. What could be the reason for this?
We thank the reviewer for this relevant comment. The reasons underlying lower satisfaction with lifestyle counselling were explored qualitatively in the open-ended survey questions. Results showed that some medical specialists felt uncomfortable discussing lifestyle, particularly because they were concerned about being perceived as judgmental or damaging the patient–physician relationship. These findings are illustrated by the quotes presented in the Results. Furthermore, we added the following sentence on this reflection in the Discussion:
"Furthermore, our qualitative findings indicated that some medical specialists felt uncomfortable discussing lifestyle because they feared being perceived as judgmental."

➢ The results of Figure 7 illustrate that the medical specialists especially talk about lifestyle, but no actual concrete plans or referrals are provided. This is in line with the findings of the patients that they feel they have changed their lifestyle themselves. This is a topic for discussion: how to enable specialists to become more concrete?

How to support the patients? The LFO is a nice solution for this.
We thank the reviewer for this valuable suggestion. We agree that our findings indicate a gap between discussing lifestyle and taking subsequent actions after addressing lifestyle. While medical specialists frequently reported discussing lifestyle, both the quantitative and qualitative findings suggest that this does not consistently translate into referral or structured support, and many patients reported initiating lifestyle changes on their own. In line with the suggestion, we have expanded the Discussion, including the link between our quantitative findings on follow-up strategies and the Lifestyle Front Offices as a promising approach to bridge the gap between lifestyle discussions and undertaking subsequent actions.
"On the individual level, structural embedding of lifestyle care requires investment in the education and training of medical specialists, ensuring that lifestyle counselling and referral skills are integrated into (postgraduate) medical education. **Our quantitative findings to this that lifestyle discussions do not consistently translate into concrete referrals or follow-up, highlighting the need for clear referral pathways and structured lifestyle support.** One promising approach, addressing barriers at the inner level **and quantitative findings on subsequent actions after discussion**, is the recent introduction of so called "Lifestyle Front Offices" (LFOs) in Dutch hospitals [32]."

➢ It seems like less results are available from the patient survey. Even the questionnaire mentions 27 questions, but only 14 are shown in the supplement.
We thank the reviewer for this observation. The discrepancy is explained by the survey's adaptive structure. Several questions were repeated based on previous responses (e.g., disease, lifestyle factor and subsequent actions undertaken). As a result, the questionnaire consisted of fewer unique questions than the total number of question screens. We have clarified this in the Supplementary Materials as follows:
** **Note**: The survey was designed with conditional branching and repeated items. Participants were asked follow-up questions for each relevant disease, lifestyle factor and subsequent actions undertaken, depending on their previous responses. Therefore, although the survey contained 27 question screens, these were based on 14 unique questions, which are presented below.*

➢ The figure in the supplement is labeled opposite to the figures in the main paper (inconvenient with respect to reading).
We thank the reviewer for this helpful suggestion. We have revised the supplementary figure to ensure consistency with the figures in the main manuscript. The response categories are now presented in the same left-to-right order, and the colour scheme has been aligned to improve readability.

VERSION 2 - REVIEW

**Reviewer**	**1**
**Name**	**Asiri, Faris Y.**
**Affiliation**	**Department of Preventive Dental Sciences, College of Dentistry, King Faisal University**
**Date**	**28-Jul-2026**

COI

The authors have addressed my previous comments. The revised manuscript has improved the reporting and overall transparency. The additional methodological details strengthen the manuscript. I have no further comments.
