# Supplementary material for: Implementation of lifestyle recommendations in the Dutch medical specialists’ guidelines: a mixed-methods cross-sectional survey study
Source: BMJ Open. 2026 Aug 10;16(8):e120063. doi: 10.1136/bmjopen-2026-120063 (PMC13475326; doi:10.1136/bmjopen-2026-120063)
Supplement: Supplementary file 2 [file bmjopen-16-8-s001.docx]

**Supplementary material**

**Figure S1**. **Flow diagram of medical specialists included in the study**

**Figure S2**. **Flow diagram of patients included in the study**

**File 1. Healthcare professional survey.** Survey assessing implementation of lifestyle recommendations from clinical practice guidelines (healthcare professional perspective).

**Part 1 – Informed consent**

The aim of this survey is to assess the extent to which lifestyle recommendations from clinical practice guidelines are implemented in current healthcare practice among general practitioners, physiotherapists, and medical specialists. Lifestyle recommendations refer to advice or recommendations regarding lifestyle (interventions) related to physical activity, smoking, alcohol and substance use, nutrition, relaxation/stress, and sleep. This survey was developed and conducted by the Knowledge Institute of the Dutch Association of Medical Specialists on behalf of the Lifestyle in Healthcare Consortium. By completing this survey, you consent to the anonymous use of your responses for research and publication purposes. No personal data will be stored. The survey takes approximately 10 minutes to complete.

1. Do you consent to the anonymous use of your responses?

- Yes → proceed to Part 2

No → By completing this questionnaire, you consent to the anonymous use of your responses for (scientific) publications on this topic. Data will be stored for a maximum of 10 years according to research guidelines. No personal identifying information will be stored, only your answers.

**Part 2 – General questions (context)**

2. Do you treat patients with one or more of the following conditions (cancer, cardiovascular diseases, respiratory diseases, obesity, diabetes mellitus, rheumatic diseases, dementia, skin diseases, depression)?

- Yes → proceed to Question 3
- No → end of the survey

3. Which of these conditions do you treat? (Multiple answers possible)

- Cancer
- Cardiovascular diseases
- Respiratory diseases
- Obesity
- Diabetes mellitus
- Rheumatic diseases
- Dementia
- Skin diseases
- Depression

4. In which province do you work (Groningen, Friesland, Drenthe, Gelderland, Flevoland, North Holland, South Holland, Overijssel, Zeeland, Utrecht, North Brabant, Limburg)?

5. What is your gender?

- Male
- Female
- Other
- Prefer not to say

6. I work as a:

- General practitioner
- Physiotherapist
- Medical specialist
- Resident physician (in specialist training)
- Other

7. In which healthcare setting do you work?

- Independent practice or health centre
- Long-term care institution
- Hospital (academic, teaching, general)
- Other

8. How long have you been working as a healthcare professional?

- 0–5 years
- 6–10 years
- 11–20 years
- >20 years

## **Part 3 – Implementation of lifestyle advice in clinical practice**

The following questions refer to the implementation of lifestyle advice among patients with one or more of the following conditions: cancer, cardiovascular diseases, respiratory diseases, obesity, diabetes mellitus, rheumatic diseases, dementia, skin diseases, and/or depression.

9. For how many of your patients do you perform the following actions related to lifestyle?

- Raising awareness of lifestyle
- Developing a lifestyle improvement plan together with the patient
- Referring patients to another healthcare professional for lifestyle advice
- Referring patients to online lifestyle information
- Referring patients to a combined lifestyle intervention programme (GLI)
- Referring patients to a lifestyle care coordination service
- Other, …

Response options per action:

- With (almost) all patients
- With the majority of patients
- With about half of the patients
- With a small group of patients
- (Almost) never

10. To what extent are the following lifestyle topics discussed in your practice?

- Physical activity
- Smoking
- Alcohol and substance use
- Nutrition
- Relaxation/stress
- Sleep
- Other

Response options identical to Question 9.

**Part 4 – Knowledge and experience with lifestyle recommendations from guidelines**

11. Are you aware of lifestyle recommendations from clinical practice guidelines in your field?

- Yes → proceed to Question 12
- No → proceed to Part 5

12. Do you use these lifestyle recommendations in clinical practice?

- Always
- Often
- Sometimes
- Almost never
- Never

13. Do these guidelines help you provide lifestyle advice to your patients?

- Yes, please explain
- No, please explain

**Part 5 – Actionability of guideline recommendations**

Below are examples of lifestyle-related recommendations derived from clinical practice guidelines (adapted for this survey).

Please focus on the wording of the recommendation, not its specific content:

*1. Advise patients to go outside daily, eat a healthy diet, and maintain social contacts.*

*2. Smoking, poor mental health status, and alcohol use are associated with worse outcomes.*

*3. Coordination of training and nutritional intake can be achieved through collaboration with a dietitian.*

*4. Refer patients to reliable patient information websites.*

*5. Consider informing the patient about the importance of healthy nutrition, a regular eating pattern, and the relationship between energy intake and expenditure.*

14. To what extent do these recommendations provide clear guidance for action in your clinical practice?

- Highly applicable, because …
- Applicable, because …
- Moderately applicable, because …
- Poorly applicable, because …
- Not applicable at all, because …

15. What do you primarily need to increase your ability to act on lifestyle advice?

- Continuing education or training
- More consultation time with patients
- Patient education materials (online)
- Referral options (e.g., lifestyle care coordination service or combined lifestyle intervention)
- I already have sufficient tools to address lifestyle in my practice
- Other

16. How important do you consider discussing lifestyle with your patients?

- Very important
- Important
- Neutral
- Not important
- Not important at all

17. How satisfied are you with how you discuss lifestyle with your patients?

- Very satisfied
- Satisfied
- Neutral
- Dissatisfied
- Very dissatisfied

18. How pleasant do you find discussing lifestyle with your patients?

- Very pleasant
- Pleasant
- Neutral
- Somewhat unpleasant
- Very unpleasant

**Closing**
Thank you for completing this survey.

**File 2. Patient survey.** Survey assessing implementation of lifestyle recommendations in clinical practice (patient perspective).

**Part 1 – Informed consent**

The aim of this questionnaire is to assess the extent to which healthcare professionals discuss lifestyle with patients. Examples include smoking cessation, healthy nutrition, and physical activity. This questionnaire was distributed on behalf of the Lifestyle in Healthcare Coalition, a collaboration of organizations active in and around healthcare. The Knowledge Institute of the Dutch Association of Medical Specialists is responsible for collecting and analysing the responses. The Knowledge Institute supports medical specialists in their professional practice by integrating scientific evidence and clinical experience. By completing this questionnaire, you consent to the anonymous use of your responses for (scientific) publications on this topic. Data will be stored for a maximum of 10 years according to research guidelines. No personal identifying information will be stored, only your answers. This questionnaire consists of a maximum of 27 questions* and takes approximately 7 minutes to complete.

**** Note****: The survey was designed with conditional branching and repeated items. Participants were asked follow-up questions for each relevant disease, lifestyle factor and subsequent actions undertaken, depending on their previous responses. Therefore, although the survey contained 27 question screens, these were based on 14 unique questions, which are presented below.*

1. Do you consent to the anonymous use of your responses?

- Yes → proceed to Part 2
- No → end of survey

**Part 2 – General questions (context)**

2. What is your gender?

- Male
- Female
- Other
- Prefer not to say

3. What is your age?

- <18
- 18–39
- 40–59
- 60–69
- 70+

4. In which province do you live (Groningen, Friesland, Drenthe, Gelderland, Flevoland, North Holland, South Holland, Overijssel, Zeeland, Utrecht, North Brabant, Limburg)?

5. In the past year, have you had contact with a healthcare professional for one or more of the following conditions?
*Cancer, cardiovascular diseases, respiratory diseases, rheumatic diseases, skin diseases, severe overweight/obesity, dementia, diabetes, depression*

- Yes → proceed to Question 6
- No → end of survey

6. For which of the following conditions have you received treatment? (Multiple answers possible)

- Cancer
- Cardiovascular diseases
- Respiratory diseases
- Rheumatic diseases
- Skin diseases
- (Severe) overweight/obesity
- Diabetes
- Dementia
- Depression

**Part 3 – Experiences and satisfaction with lifestyle discussions**

The following questions refer to your experiences with the treatment of the conditions listed above.

7. In the past six months, have you had contact with a physician in the hospital (medical specialist)?

- Yes → proceed to Question 8
- No → end of survey

8. What type of physician did you consult?
*For example: internist, cardiologist, pulmonologist, rheumatologist, rehabilitation physician, etc.*

9. Was lifestyle discussed during the consultation?

- Yes → proceed to Question 10
- No → end of survey
- I do not know → proceed to Question 10

10. Which topics were discussed? (Multiple answers possible)

- Physical activity
- Smoking
- Alcohol
- Nutrition
- Relaxation
- Sleep
- I do not know
- Other

11. What happened afterwards?

- I continued improving my lifestyle independently
- The healthcare professional developed a lifestyle-related treatment plan with me
- I was referred to:
  - Combined lifestyle intervention programme (GLI)
  - Lifestyle care coordination service
  - Physiotherapist
  - Dietitian
  - Nurse
  - Practice nurse at the GP practice
  - Other
- Other outcome

12. How important do you think it is that hospital physicians discuss lifestyle with you?

- Very important
- Important
- Neutral
- Not important
- Not important at all

13. How satisfied are you with the way hospital physicians discussed lifestyle with you?

- Very satisfied
- Satisfied
- Neutral
- Dissatisfied
- Very dissatisfied

14. How pleasant do you find discussing lifestyle with hospital physicians?

- Very pleasant
- Pleasant
- Neutral
- Somewhat unpleasant
- Very unpleasant

**Closing**
Thank you for completing this survey.

**Figure S3**. **Applicability of five distinctly formulated recommendations**. Results were rated by medical specialists from highly applicable to not applicable at all on a 5-point Likert scale. The percentages displayed on the left side of the plot represent the two least affirmative response options: *poorly applicable* and *not applicable at all*. The centre of the plot reflects the neutral category: *Moderately applicable.* On the right side, the percentages correspond to the two most affirmative categories: *highly applicable* and *applicable*.

**Figure S4. Subsequent actions taken after addressing lifestyle during consultations, according to patients**. The x-axis shows the number of patients who indicated which follow-up strategy was applied. The bar representing referrals is subdivided into specific referral destinations, as detailed in the legend. Percentages indicate the proportion of patients endorsing each lifestyle factor. Totals exceed 100% because patients could select multiple factors.
